# Characteristics of the Content and Variability of Dietary Fiber Components and Alkylresorcinols of Rye Grain (*Secale cereale* L.)

**DOI:** 10.3390/molecules30142994

**Published:** 2025-07-16

**Authors:** Anna Fraś, Magdalena Wiśniewska, Dariusz R. Mańkowski, Marlena Gzowska

**Affiliations:** Plant Breeding and Acclimatization Institute—National Research Institute, Radzików, 05-870 Błonie, Poland; a.fras@ihar.edu.pl (A.F.); m.wisniewska@ihar.edu.pl (M.W.); d.mankowski@ihar.edu.pl (D.R.M.)

**Keywords:** alkylresorcinols, arabinoxylans, β-glucan, lignin, rye

## Abstract

Rye (*Secale cereale* L.) is one of the most important cereals cultivated in Central and Eastern Europe, valued for its high resistance to environmental stress and high levels of bioactive compounds, such as dietary fiber (DF) and alkylresorcinols (ARR). The aim of the study was to evaluate the content and variability of DF fractions and ARR in rye grain of hybrid and population cultivars. The research was conducted on grain from four rye cultivars cultivated in five locations over three consecutive growing seasons. The content of DF, its fractions, and ARR, was determined using enzymatic–gravimetric and colorimetric methods. The results showed significant variability in all analyzed traits, with environmental conditions and G×E interaction having the greatest impact on their content. Hybrid cultivars were characterized by a higher and more stable content of bioactive compounds. Notable average values for hybrids vs. populations included DF: 153.9 vs. 151.7 g kg^−1^, NSP: 129.4 vs. 127.7 g kg^−1^, lignin: 24.5 vs. 24.0 g kg^−1^, β-glucan: 21.7 vs. 20.6 g kg^−1^, and ARR: 1015 vs. 987 g kg^−1^. The KWS Serafino cultivar characterized by the highest and most stable content of bioactive compounds. Selecting genotypes with stable chemical profiles regardless of environmental conditions is crucial for developing nutritionally valuable rye-based products.

## 1. Introduction

Rye (*Secale cereale* L.), alongside wheat, is the main cereal species grown in Central and Eastern European countries. Of all winter cereals, rye has the least soil requirements. It is distinguished by its frost hardiness, resistance to drought stress and resistance to fungal diseases. In addition, it is recommended for organic cultivation, and has a high yield, even in light soils [1].

At the end of the 20th century, the first hybrid rye cultivars were developed in Germany through breeding progress. Unlike population cultivars, these hybrids were characterized by a higher yield potential. The rapid development of this new direction in rye breeding contributed to the popularization of hybrid varieties, which now account for about 80% of the total rye crop [2]. In addition to their greater yield potential, hybrid rye, compared to population rye, is distinguished by better uniformity, greater drought tolerance, and a modified chemical composition [2,3,4]. The reduced content of anti-nutritional compounds in hybrid rye grain has made it possible to use it in the diets of monogastric animals [5]. Furthermore, ongoing breeding work aims to improve rye grain quality by changing the protein and starch content as well as the thousand kernel weight and ultimately enhancing the technological value of rye flour. Another important breeding goal is to improve grain health by increasing resistance to fungal diseases such as ergot and Fusarium head blight [6].

Rye is an important crop used for food production, animal feed, ethanol, and as a substrate in biogas plants. Its intended use is determined by the chemical profile of the grain, which, in addition to nutrients, contains a variety of compounds with health-promoting properties. Among this group of compounds are dietary fiber and alkylresorcinols. Due to their concentration in the outer layers of the grain, consuming products made from whole rye grain is preferred—such as bread, crispbread, pasta, flakes, sweet and salty snacks, and chips. Including wholegrain rye products in the daily diet is associated with a number of well-documented health benefits, such as reducing the risk of various cancers, diabetes, coronary heart disease, lowering cholesterol levels, supporting weight regulation, and protecting intestinal microbiota [7,8]. Unfortunately, the consumption of wholegrain rye products remains insufficient, which may be due to the public′s lack of nutritional awareness.

According to the definition presented by the Codex Alimentarius Commission (CAC), dietary fiber includes carbohydrate polymers with ten or more monomeric units that are not hydrolyzed by endogenous enzymes in the human small intestine. Chemically, the dietary fiber of cereal grains is a complex in which non-starch polysaccharides account for the largest share. In rye grain, these are primarily arabinoxylans and, in small amounts, β-glucan. In addition, an important part of the fiber in cereals is lignin [9,10,11]. Dietary fiber is divided into soluble and insoluble fractions, each of which exhibits different physiological effects. Rye grain is distinguished from other cereal species by its significantly higher content of the soluble fraction, which includes soluble NSP fractions such as arabinoxylans and β-glucan [12]. This fraction has the ability to form viscous solutions in an aqueous environment, so it is a major contributor to the reduction in postprandial blood glucose and insulin. Furthermore, it slows down the passage of food through the stomach, improves digestibility and absorption and increases the feeling of satiety. In the large intestine, the soluble fraction is fermented by the microflora into short-chain fatty acids [13,14,15]. In cereal grains, the insoluble fiber fraction consists of cellulose, insoluble NSP fractions and lignin. This fraction is not hydrolyzed by digestive enzymes in the human gastrointestinal tract but partially digested by microflora of the large intestine. It is characterized by passive water binding properties, which increase stool bulk and frequency and decrease intestinal transit time. This fraction also has the ability to form complexes with carcinogenic substances, whereas lignin binds a substantial amount of bile acids and cholesterol [13,14,15].

Rye, besides dietary fiber, is also a source of other bioactive components, such as sterols, phenolic acids, tocols and folates. It is also the most abundant source of alkylresorcinols (5-n-alkylresorcinols, resorcinol lipids, ARR) among cereals. These compounds belong to the class of non-isoprenoid phenolic lipids, which are derivatives of 1,3-dihydroxy-5-methylbenzene and are characterized by an odd-carbon side chain substituted at position 5 of the benzene ring [16]. The solubility of these compounds, and consequently their bioactivity, differs between the different ARR homologs, with the long-chain ones dissolving more slowly [12]. ARRs are considered compounds that determine a number of potential health benefits. Among other things, they influence the lowering of intestinal cholesterol levels, increase glucose tolerance and insulin sensitivity, exhibit anticancer effects by inhibiting the growth of colon cancer cells, and have beneficial effects on body weight regulation [12,17,18]. For this reason, it is important to continuously promote the consumption of wholegrain cereal products. Due to the distribution of ARRs in the outer parts of the kernel, these compounds have been proposed as potential biomarkers of wholegrain product consumption [12,16].

Rye products made from whole grain may be the answer to the growing interest in foods with simple ingredients and health-promoting properties. In order to continuously increase their market share, there is a need for ongoing promotion of rye varieties that, in addition to favorable agronomic traits, will also stand out for their stability under different environmental conditions and the best possible chemical composition. Therefore, the aim of this study was to assess and characterize the content of dietary fiber and its components, as well as alkylresorcinols, in hybrid and population rye varieties, and to examine their variability. To investigate the influence of genotype, environment, and their interaction on the content of the analyzed components, the varieties were cultivated in different locations over three consecutive growing seasons. This research topic arises from the limited and inconsistent literature data regarding differences in the chemical composition of rye grain from population and hybrid varieties. Furthermore, the insufficient availability of up-to-date research results on the variability and stability of the chemical profile of rye grain under changing environmental conditions justifies the need for continued studies in this area.

## 2. Results and Discussion

The content of individual chemical components in rye grain differed significantly, between the studied cultivars, years and sites of cultivation. The mean contents of each component and the results of the analysis of variance are presented in Table 1. Rye grain is the richest source of dietary fiber among cereals. DF is a complex compound, so its variability depends on the proportion of individual components [13]. The main constituent of dietary fiber of rye grain is non-starch polysaccharides (NSP), more than 80%, especially the insoluble fraction (I-NSP), which accounts for about 55% of the DF.

Rye grain contains almost three times the content of the soluble fraction of NSP (S-NSP), compared to wheat and triticale grain. This fraction is most responsible for the viscous properties of dietary fiber in cereal grains, with an average content of 43.8 g kg^−1^ for rye, 14.1 g kg^−1^ for wheat and 18.0 g kg^−1^ for triticale [19]. Hybrid rye cultivars were distinguished by significantly higher NSP content compared to population cultivars, including the traditional one, with average contents of 128.9 g kg^−1^ and 129.9 g kg^−1^ for hybrid cultivars and 127.6 g kg^−1^ and 127.7 g kg^−1^ for the other two (Dańkowskie Rubin and Rye NN). In the case of I-NSP, the cultivar KWS Binntto contained the highest amount of this polysaccharide fraction (86.5 g kg^−1^), while in the grain of the second of the hybrid cultivars, KWS Serafino, the amount of I-NSP was significantly lower (84.2 g kg^−1^) and at the same time similar to the content determined in the grain of population cultivars. Nevertheless, the high value of the correlation coefficient at r = 0.88 confirms the significant relationship between NSP and I-NSP in the studied rye cultivars. No such relationship was found for the content of the S-NSP fraction. It was observed that hybrid cultivars KWS Binntto and KWS Serafino contained extreme contents of this fraction, at the level of 42.4 g kg^−1^ and 45.7 g kg^−1^, respectively. Population ryes did not differ in the amount of S-NSP, which averaged 43.5 g kg^−1^. The greatest variation in the studied material in terms of S-NSP content occurred between the growing seasons, with average contents ranging from 121.5 g kg^−1^ for the 2016/2017 season to 134.5 g kg^−1^ for the 2017/2018 season. As with the analysis of the chemical composition of the cultivars, there were also directly proportional relationships between the content of S-NSP and the insoluble fraction of these polysaccharides between the growing seasons. No such relationship was observed for the S-NSP fraction, which probably explains the results presented in Table 2. On the basis of the analysis of variance, it was shown that the contents of NSP and I-NSP were determined to the greatest extent among all the analyzed traits by environmental conditions, 62.0% and 74.5%, respectively, and by the G×E interaction, while the content of the S-NSP fraction was much more determined than the others by genotype, at 8.5%, which may have influenced the lack of a direct proportion between the amount of this fraction and the total NSP content. Based on the data described in Table 2, it can be concluded that all analyzed traits were determined to the greatest extent by environmental conditions and G×E interaction. In the former case, this variation ranged from 39.8% for lignin to 74.5% for NSP, while in the latter case it ranged from 22.0% for I-NSP to 58.0% for lignin.

The average content of NSP in the different cultivation localities also varied, with the Prusim locality standing out the most in terms of this feature, for which the NSP content was found to be 132.0 g kg^−1^. For this locality, the content of the I-NSP and S-NSP fractions also had the highest values, at 87.1 g kg^−1^ and 44.9 g kg^−1^, respectively (Table 1). Bach Knudsen [20] also described the content of NSP and the S-NSP fraction in rye grain and for the first parameter obtained values higher than those described above, at 147.0 g kg^−1^, while the compactness of S-NSP was lower in the author’s study, at 37.6 g kg^−1^. A similar study was conducted by Boros et al. [19], who in both cases obtained lower values compared to those described, at the level of 121.0 g kg^−1^ for NSP and 37.0 g kg^−1^ for S-NSP, while the content of the I-NSP fraction the study authors determined was at a comparable level of 84.0 g kg^−1^. The content of NSP in rye grain was also determined by Bach Knudsen [7], who reports an average amount of these compounds at 185.0 g kg^−1^, a significantly higher value compared to the described studies. Slightly higher results for the content of NSP and its individual fractions were also described by Arczewska-Włosek et al. [21], who obtained the following densities: NSP—155 g kg^−1^, I-NSP—100.0 g kg^−1^, S-NSP—55.0 g kg^−1^.

According to the definition of dietary fiber [10], NSP includes cellulose and non-cellulosic polysaccharides (NCPs). This group of compounds in the case of cereal grains primarily includes arabinoxylans (AX) and β-glucan, in the case of rye grain, AX are the main polymers in the cell wall. In the work presented here, AX accounted for about 56% of the total NSP content, of which 20% were water-extractable AX and 36% were water-unextractable AX. There were no significant differences in AX content between the studied rye cultivars (Table 1), and the average amount of these compounds was at the level of 72.1 g kg^−1^. Significant differences were found between years and cultivation localities. As in the case of NSP, the extreme compactness of AX was obtained for crop years 2016/2017 and 2017/2018, at the level of 69.8 g kg^−1^ and 75.0 g kg^−1^, respectively. The AX content in each growing region ranged from 70.4 g kg^−1^ for Walewice to 74.9 g kg^−1^ for Prusim (Table 1). As a key component of NSP, AX content was significantly correlated with both their total content and individual fractions, and the correlation coefficients were r = 0.87 for NSP, r = 0.67 for I-NSP and r = 0.30 for S-NSP, respectively. Consistently, the AX content was mainly determined by environmental conditions and G×E interaction, 57.9% and 35.0%, respectively (Table 2). The variability in AX content in the tested material, obtained for individual variables, is consistent with the literature data described by Kucerova [5]. The author tested hybrid and population rye cultivars for AX content, and the relationships obtained were similar to those described above. The amount of AX determined by Kucerova in hybrid rye grain was on average 80.4 g kg^−1^, and in population rye grain 78.8 g kg^−1^. Thus, these values are slightly higher than those obtained in the presented work. Other authors in their studies described higher AX content in rye grain in the range from 85.5 g kg^−1^ to 96.0 g kg^−1^ [7,21,22]. Subsequent authors [23,24] described an even wider range of AX content in rye grain, from 71.0 g kg^−1^ to 122.0 g kg^−1^, while Cyran and Łapiński [25] determined the average content of these compounds at the level of 85.2 g kg^−1^.

The second key component of dietary fiber and NSP in cereal grain is β-glucan. This compound has health-promoting properties, which are the best documented of all fiber components [26]. Rye grain is characterized by the third, highest content of this component after oats and barley. The rye genotypes studied differed significantly in terms of β-glucan content, ranging from 20.2 g kg^−1^ for Rye NN to 21.8 g kg^−1^ for KWS Serafino, with hybrid cultivars characterized by a significantly higher content compared to the other two. Significant differences in β-glucan content were also observed in subsequent crop years, ranging from 20.1 g kg^−1^ for the 2016/2017 season to 21.9 g kg^−1^ for the 2018/2019 season. The average β-glucan content of each location also varied significantly, although slightly, with values ranging from 20.5 g kg^−1^ for Wyczechy to 21.9 g kg^−1^ for Walewice (Table 1). Of all the chemical components analyzed, β-glucan content was the feature most determined by genotype, at 10.9% (Table 2), followed by environment and G×E interaction, at 41.6% and 42.2%, respectively. This result may indicate little variation in the content of this component in changing environments. The amount of β-glucan in rye grain was also described by Bach Knudsen [20], who reports an average content of this component at the level of 17.0 g kg^−1^. In the studies described by other authors, the β-glucan content in rye grain was characterized by wide variation, and the values obtained ranged from 10.6 g kg^−1^ to 23.0 g kg^−1^ [23,27]. A β-glucan content comparable to those described above, at 21.0 g kg^−1^, was obtained by Kołodziejczyk et al. [22], who studied its content in whole rye flour.

An important part of the dietary fiber of rye grain is the insoluble fraction. The second component of this fraction after I-NSP is lignin. This is a parameter whose content was determined to the least extent by genotype (0.4%) of all the analyzed traits, and to the greatest extent by G×E interaction (58.0%) (Table 2). The obtained amount of lignin was at the same time the most diverse parameter, and the coefficient of variation of this trait was about 10%. The content of this trait in the analyzed rye cultivars ranged from 23.9 g kg^−1^ for Rye NN to 24.5 g kg^−1^ for the KWS Serafino cultivar. Similarly to NSP and AX, hybrid cultivars were characterized by significantly higher lignin content compared to other genotypes. Over the three years of cultivation, the highest average content of this component, at the level of 26.9 g kg^−1^, was obtained in the 2016/2017 season, and the lowest content at the level of 22.2 g kg^−1^ in the 2018/2019 season. Cultivation locations also differed in the average lignin content, although to a lesser extent than in the case of cultivation seasons. The lowest average content of this component at 23.9 g kg^−1^ was found in Prusim, and the highest at 26.7 g kg^−1^ in Walewice (Table 1). Lignin content was significantly negatively correlated with the content of NSP (r = −0.36), I-NSP (r = −0.42) and AX (r = −0.32). The obtained lignin contents were within the range obtained by Nystrom et al. [28] for different rye cultivars, both population and hybrid. However, these authors obtained greater variability for this parameter, and the values ranged from 20.0 g kg^−1^ to 29.0 g kg^−1^. Boros et al. [19] in their studies conducted for 18 rye cultivars obtained similar, although slightly higher lignin contents, ranging from 26.0 g kg^−1^ to 30.0 g kg^−1^, while Bach Knudsen et al. [7] reported the content of this component in whole rye grain at the level of 17.0 g kg^−1^, which is significantly lower than the result described in this study.

Dietary fiber content in the tested material was calculated as the sum of the insoluble and soluble fractions of NSP and lignin. The analyzed genotypes were divided into two statistically significantly different groups in terms of DF content. The first group consisted of hybrid cultivars, characterized by a higher DF content, at the level of 153.3 g kg^−1^ and 154.4 g kg^−1^, respectively, for the KWS Binntto and KWS Serafino cultivars. The Dańkowskie Rubin and Rye NN cultivars contained an average of 151.7 g kg^−1^ DF (Table 1). The DF content in the individual years of cultivation differed significantly, and the observed relationships concerned the content of NSP and I-NSP. These relationships are also confirmed by high significant correlations obtained between DF content and NSP (r = 0.89), I-NSP (r = 0.74), AX (r = 0.76), as well as slightly lower, but still significant correlations with the content of S-NSP (r = 0.22) and β-glucan (r = 0.19). Among the individual cultivation locations, the cultivars grown in only one location, Prusim, differed significantly in DF content and were characterized by the highest amount of this component at the level of 155.9 g kg^−1^. The material grown in the remaining locations did not differ significantly and contained an average DF content of 152.0 g kg^−1^ (Table 1). The DF content was determined in 51.7% by environmental conditions and in 40.9% by the G×E interaction (Table 2). The dependence of this parameter on genotype was only 2.3%, which most likely resulted from the relatively high share of genotype in the variability of soluble fiber fractions, i.e., S-NSP and β-glucan (Table 2). The DF content in rye grain has been described in the literature by various authors, and the obtained values fall within a wide range, which may result from the diversity of the research material and the use of different analytical methods. The most comparable results to those obtained in this study were described by Boros et al. [19], who, using an analogous method of determination, obtained the DF content in rye grain at an average level of 149.0 g kg^−1^, ranging from 140.0 g kg^−1^ to 164.0 g kg^−1^. Many authors, describing the DF content, calculated its amount as the sum of the soluble fraction (SDF) and insoluble fraction (IDF), determined as a whole. The content of DF and its fractions was described in the study by Ikram et al. [29], who examined very diverse material and obtained DF contents ranging from 168.9 g kg^−1^ to 244.7 g kg^−1^, including the IDF ranging from 145.3 g kg^−1^ to 178.0 g kg^−1^, and the SDF from 23.6 g kg^−1^ to 66.7 g kg^−1^. Other authors [25] characterized the DF content in the population rye grain at 176.0 g kg^−1^, including 128.0 g kg^−1^ of the IDF and 48.0 g kg^−1^ of the SDF, while Kołodziejczyk et al. [22] obtained contents of 184.0 g kg^−1^, 145.0 g kg^−1^ and 39.0 g kg^−1^ for the same features, respectively. The high contents of DF and its IDF described by the above researchers result mainly from the applied determination method, in which the IDF includes a larger group of compounds than in the above-mentioned studies, including lignin and related substances, as well as some lipid compounds, which directly affects the higher final result for this fraction. The contents of the SDFs presented in these studies were always similar to the results obtained in this work for the contents of the S-NSP fraction.

Rye is the richest source of alkylresorcinols (ARR) among cereals, which are compounds with many health-promoting properties. Due to the location of ARR in the outer layers of the grain, they play the greatest role in wholegrain rye products. The ARR content in the rye cultivars studied differed significantly between genotypes, years of cultivation, and locations (Table 1). The highest average content of these compounds at the level of 1037 mg kg^−1^ was found in the grain of the hybrid cultivar KWS Binntto, and the lowest 961 mg kg^−1^ in the grain of the population Rye NN. Among the analyzed growing seasons, only the 2018/2019 season was distinguished by a significantly higher ARR content at the average level of 1103 mg kg^−1^. The ARR content in individual cultivation locations ranged from 969 mg kg^−1^ for cultivars grown in Boguszyn to 1027 mg kg^−1^ for genotypes originating from Wyczechy. Similarly to the content of DF and its components, the content of ARR was determined to the greatest extent by environmental conditions (58.9%), then by the G×E interaction (26.7%), and to the least extent by genotype (7.1%) (Table 2). According to some literature data, hybrid rye cultivars contain a reduced amount of antinutritional substances, including ARR, which is important from the perspective of animal nutrition [30]. In the case of the results described in this work, such relationships did not occur, and the results obtained, regardless of the type of rye, oscillated at an average level of about 1000 mg kg^−1^. Milczarek et al. [31] report in their study a much lower content of alkylresorcinols determined in hybrid rye cultivars, at the level of 655.75 mg kg^−1^. Sułek et al. [32] analyzed the ARR content in hybrid and population cultivars of rye and also obtained results lower than those described above, ranging from 533 mg kg^−1^ to 662 mg kg^−1^. These authors, based on research conducted over two consecutive growing seasons, obtained higher ARR contents for hybrid cultivars than for population cultivars. In addition, in their research, they explained the effect of unfavorable weather conditions during plant growing season, especially high level of rainfall on the increase in ARR content in rye grain. Similar research was conducted by Grabiński et al. [33], who also obtained ARR contents in a narrow range from 535 mg kg^−1^ to 563 mg kg^−1^, with the highest content of these compounds being characteristic of the hybrid cultivar. Much higher ARR contents in rye grain in the range from 1105 mg kg^−1^ to 1152 mg kg^−1^ were obtained in their research by Kulawinek et al. [34]. Nystrom et al. [28] analyzed the ARR content in ten rye cultivars and obtained values at an average level of 1030 mg kg^−1^, ranging from 797 mg kg^−1^ to 1231 mg kg^−1^, while Boros et al. [19] analyzed 18 genotypes and determined the average ARR content at the level of 815 mg kg^−1^. Such a large range in the ARR content in rye grain, as described by different researchers, may be caused by the high sensitivity of this trait to changing environmental conditions, and only to a small extent by a genetic factor.

**Figure 1:**
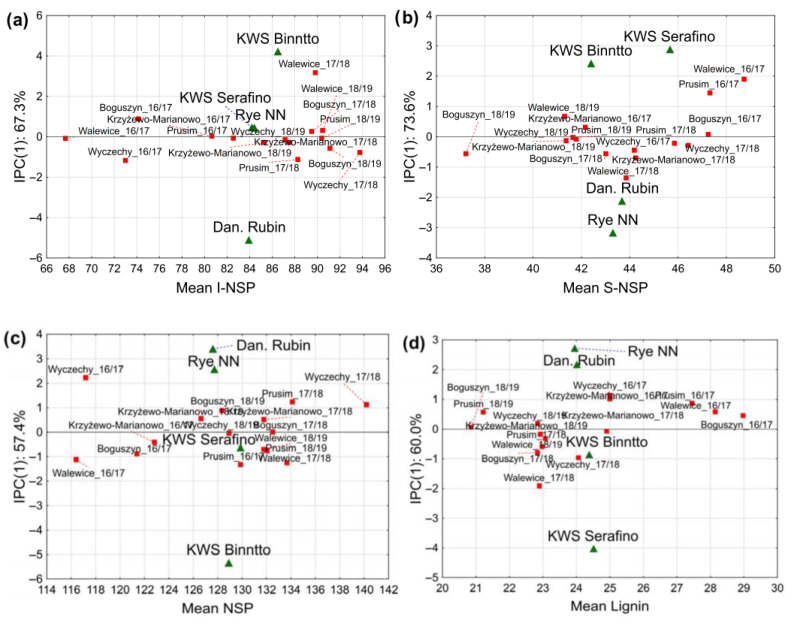

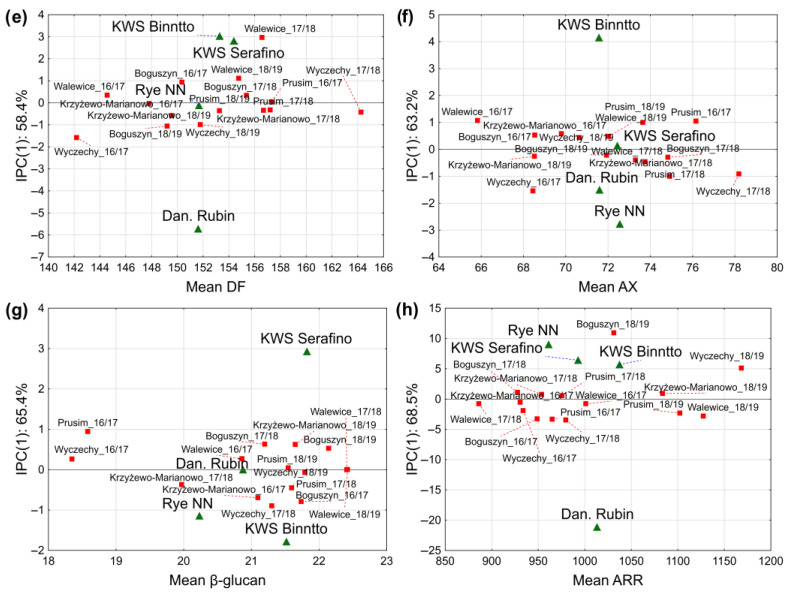
Biplots from AMMI analysis for the mean content of the following: (**a**) I-NSP; (**b**) S-NSP; (**c**) NSP; (**d**) Lignin; (**e**) DF; (**f**) AX; (**g**) β-glucan; (**h**) ARR vs. IPC (1). The percentage value given for IPC1 indicates its contribution to the total explained interaction variance.

Due to the very high contribution of the environment (E) and the G×E interaction in the variability of all the analyzed parameters, the AMMI analysis (Additive Main effects and Multiplicative Interaction model) was performed. The AMMI model combines ANOVA (main effects) and PCA (interaction effects) to analyze genotype (G) × environment (E) interactions. This analysis allowed for the precise indication of the most stable rye genotypes in terms of individual traits in changing environments. Below is an interpretation of hypothetical AMMI biplots (Figure 1) for key rye components. In the case of the I-NSP content, the AMMI analysis showed statistically significant interaction effects for the genotype, environment and G×E interaction (Table 2). For all the studied parameters, the first two interactive components, IPC (1) and IPC (2) (Interaction Principal Component), were responsible for most of the interactions. In the case of the I-NSP content, the IPC (1) component was responsible for 67.3% of the variability, and the IPC (2) component for 27.4% of the interaction. Based on Figure 1a, it can be concluded that in terms of I-NSP content, two cultivars, KWS Serafino and Rye NN, can be considered stable, while the cultivars KWS Binntto and Dańkowskie Rubin were characterized by a clear interaction effect with environmental conditions. In terms of environmental conditions, the greatest interactions were found for the Walewice location in the 2017/2018 season, which was responsible for the interactive effect for the above cultivars.

For the S-NSP content, significant interactive effects were found for all variables and the F-statistic values are presented in Table 2. The share of the IPC (1) and IPC (2) components in shaping the interactive variability was 73.6% and 18.8%, respectively. All the rye genotypes tested showed an interactive effect for the S-NSP content (Figure 1b), which was influenced primarily by the conditions in the Prusim and Walewice localities in the 2016/2017 season. For the total NSP content, similarly to the individual fractions, significant interactive effects were found for all variables, while the share of the individual components in their variability was 57.4% for IPC (1) and 32.8% for IPC (2). In terms of NSP content, only the hybrid cultivar, KWS Serafino, showed stability, while the remaining three genotypes showed a clear interactive effect (Figure 1c). The location that provided the most favorable conditions for the emergence of G×E interaction effects for this parameter was Wyczechy in the 2017/2018 season. In relation to lignin content, the IPC (1) component accounted for 60% of the interactive variability, and the IPC (2) component for 23.4% of the variability. Moreover, similarly to the other traits, statistically significant interaction effects were found for all environmental variables (Table 2). Among the analyzed genotypes, both population rye cultivars and the hybrid cultivar KWS Serafino were characterized by a significant interactive effect in terms of lignin content. It was mostly influenced by the conditions in Wyczechy in the 2016/2017 season and Walewice in the 2017/2018 season (Figure 1d). The hybrid cultivar KWS Binntto was characterized by stability in terms of lignin content in various environmental conditions. AMMI analysis for dietary fiber (DF) content also showed a significant interactive effect in the variability for the G, E and G×E interaction (Table 2). The IPC (1) and IPC (2) components accounted for 58.4% and 28.1% of the interactive variability, respectively. In terms of DF content, only the traditional rye cultivar Rye NN was characterized by stability in variable environmental conditions (Figure 1e). The remaining cultivars Dańkowskie Rubin, KWS Serafino and KWS Binntto were characterized by a clear interactive effect with environmental conditions, which were determined primarily by the conditions in Walewice in the 2017/2018 season and, to a lesser extent, in Wyczechy in the 2016/2017 season. The interactive variability of AX content was determined by the first IPC (1) component in 63.2%, and by the second IPC (2) component in 19.7%. Similarly to the content of the remaining components, statistically significant interactive effects were found for all environmental variables. Among the analyzed genotypes, the only stable cultivar in terms of AX content was the hybrid cultivar KWS Serafino, similarly to the NSP content (Figure 1f). Similarly to NSP, the greatest influence on the interactive variability of the remaining three rye genotypes was exerted by the conditions that occurred in the Wyczechy locality in the 2016/2017 season. For β-glucan content, similarly to other parameters statistically significant interactive effects were found for all environmental variables (Table 2). The share of IPC (1) and IPC (2) components in the interactive variability for this trait amounted to 87.3% in total. Among the rye genotypes studied, only the Dańkowskie Rubin population cultivar was characterized by stability in terms of β-glucan content (Figure 1g). A characteristic feature of this cultivar was stability only in terms of β-glucan content, and in the case of the remaining analyzed parameters, this genotype showed interactive effects. Among the variable environments, the most favorable conditions for the formation of G×E interactions in relation to β-glucan content were observed for the Prusim locality in the 2016/2017 cultivation season. The last parameter of rye grain analyzed was the ARR content, for which the AMMI analysis showed significant interactive effects for all environmental variables. The share of the IPC (1) component in the interactive variability was 68.5%, while IPC (2) was 22.5%. Based on Figure 1h, it can be stated that all analyzed rye genotypes were characterized by a clear interactive effect with environmental conditions. In the case of this feature, no stability of the studied cultivars was found, and the interactive effect was caused by the conditions occurring in the localities of Boguszyn and Wyczechy in the 2018/2019 season.

## 3. Materials and Methods

### 3.1. Materials

The four Polish rye grain cultivars (*Secale cereale* L.) were supplied by the KWS Poland Ltd. Co. (Poznań, Poland). Two of them, KWS Binntto and KWS Serafino, were hybrid cultivars, and the next two, Dańkowskie Rubin and Rye NN were population cultivars. Furthermore, the Rye NN genotype was not a commercial cultivar but was traditionally cultivated by the farmer for many years. Selected cultivars were grown in five locations with different climatic conditions—Boguszyn (50°55′ N; 16°58′ E), Krzyżewo (53°02′ N; 22°76′ E); Prusim (53°88′ N; 15°48′ E); Walewice (52°06′ N; 19°69′ E); Wyczechy (53°84′ N; 16°92′ E)—and during three successive growing seasons: 2016/2017, 2017/2018 and 2018/2019. Samples for analysis were sent as bulk samples for a given genotype, from a given location in each growing season. For this reason, in the further stages of the analyses, it was assumed that the experimental design was a three-factor completely randomized design. Rye grain samples were ground prior to chemical analysis in the Cyclotec TM laboratory mill (FOSS, Hillerod, Denmark) through a 0.5 mm sieve. In total, 100 g of whole grain was ground each time. All samples were stored in a fridge in sealed plastic cups until analysis.

### 3.2. Analytical Methods

#### 3.2.1. Dietary Fiber

Dietary fiber (DF) content was determined using the enzymatic chemical method in accordance with AACC 32–25 [35] and AOAC 994.13 [36] procedures as a sum of non-starch polysaccharides (NSPs) and lignin.

##### NSP

NSP content with its fractionation to soluble (S-NSP) and insoluble (I-NSP) fractions was determined using gas chromatography (GC) according to the method of Englyst and Cummings [37]. In this procedure, the NSP of each fraction is a sum of the five individual monomers arabinose, xylose, mannose, galactose and glucose. After the enzymatic hydrolysis of starch, the samples were centrifuged and split into soluble (ethanol precipitates from supernatant) and insoluble (remaining pellet) fractions. Each of these fractions was hydrolyzed with 1 M sulfuric acid (100 °C, 2 h) to monosaccharides and converted to volatile alditol acetates. *Meso*-erythritol was used as an internal standard. In parallel with the main samples, four reference mixtures with varying concentrations of standard sugars were included at this stage of the analysis. The alditol acetates were separated on a capillary quartz column Rtx-225 (0.53 mm × 30 m) using the Clarus 600 gas chromatograph (Perkin Elmer, Waltham, MA, USA) equipped with an autosampler, splitter injection port and flame ionization detector. The carrier gas was He. Separation was performed at 225 °C, with injection and detection at 275 °C. Based on the obtained NSP results, the total arabinoxylans (AX) content was calculated.

##### Lignin

Lignin and other insoluble residues were determined gravimetrically, as described by Theander and Westerlund [38], as a dry (105 °C, 16 h) residue of a sample previously digested with 72% sulfuric acid that had been incinerated (550 °C, 5 h). The percentage contents of lignin and associated polyphenols were calculated on the basis of the loss in weight by incinerating the dried insoluble material.

#### 3.2.2. β-Glucan

β-glucan is a component of NSP, classified as a non-starch polysaccharide. Its content was analyzed by the colorimetric method, using the Megazyme procedure (Bray, Ireland) in accordance with the AACC 32–23 [35] and AOAC 995.16 [36] methods. Samples were suspended and hydrated in a buffer solution of pH 6.5, next incubated with purified lichenase enzyme and filtered. Subsequently, an aliquot of the filtrate was hydrolyzed to completion with purified β-glucosidase. The released D-glucose was determined using the GOPOD reagent. Absorbance was measured at 510 nm (UV-1601, Rayleigh, BRAIC, Beijing, China). In parallel, absorbance of different glucose concentrations, ranging from 20 to 100 μg/mL, was performed to plot the calibration curve.

#### 3.2.3. Alkylresorcinols

Alkylresorcinols (ARR) were determined by the colorimetric method according to the procedure described by Tłuścik et al. [39] with modifications. Alkylresorcinols were extracted from the intact kernels using acetone at ratio 1:4 *w*/*v*, and the Fast Blue B diazonium salt was used to develop the color. The absorbance was measured at 520 nm (UV-1601, Rayleigh, BRAIC, Beijing, China) for each sample against the reagent blank. A calibration curve was prepared from the standard solution containing 1 mg of purified 5-n-pentadecylresorcinol in 1 mL of n-propanol, of which 5, 10, 15, and 20 μL were used. The ARR content was expressed in milligrams per kilogram dry weight of the sample.

All chemical analyses were performed in duplicate and the mean value was accepted if the difference between duplicates was below 10%. All the obtained results were calculated on a dry weight basis (DW).

### 3.3. Statistical Analysis

To study the variability in the contents of dietary fiber components and alkylresorcinols, content in analyzed materials, a three-way fixed model of analysis of variance (ANOVA) was performed. For all analyzed variables, in accordance with the Lindeberg–Lévy theorem, it was assumed that their distributions are asymptotically convergent to the normal distribution. Homogeneity of variances was confirmed by Levene′s test. The results of the variance analysis were supplemented by comparing the mean values for the main effects and the interaction effect using Tukey’s multiple comparison procedure (post hoc test) at the significance level of α = 0.05. The Pearson correlation coefficients between analyzed traits were also calculated. Additionally, to describe the G×E interaction more precisely, an analysis was performed for selected variables using the AMMI model where the environments are indicated as location × growing season combinations. Detailed interpretation was performed by assessing the significance of the additive and multiplicative components of the model and the biplot for genotypic and environmental means in relation to the environmental component IPC (1). All statistical analyses were performed using Statistica (data analysis software), version 14 [40].

## 4. Conclusions

Three-year studies conducted in several locations allowed for the assessment of winter rye genotypes in terms of DF content, its components, and the amount of ARR in grain. Characteristics of the grain chemical composition and assessment of the variability of the studied genotypes also allowed for the comparison of hybrid rye cultivars with population rye. Based on the conducted studies, it can be concluded that most of the analyzed features were determined primarily by environmental conditions and the G×E interaction, which indicates a high and varied sensitivity of rye to weather conditions. The influence of genotype on the analyzed traits was much lower, below 11%, and was mostly determined by the content of β-glucan (10.9%), S-NSP (8.5%) and ARR (7.1%). Hybrid cultivars were characterized by a significantly higher content of DF and most of its components compared to population cultivars. A similar trend was not observed in the ARR content; however, the traditional Rye NN cultivar contained the least ARR and β-glucan in the kernels among the genotypes studied. Nevertheless, it did not differ in terms of the remaining analyzed components from the second population cultivar, Dańkowskie Rubin. The significant effect of the growing season and cultivation location was confirmed by the high interaction values obtained for all the studied features. The analyzed rye genotypes were characterized by the highest content of individual chemical components of grain in the 2018/2019 season. The same effect, independent of the growing season, was observed in the case of cultivation in the Prusim location. Based on the AMMI analysis, it was found that hybrid rye cultivars were characterized by stability for NSP, I-NSP, lignin and AX. In addition, the hybrid cultivar KWS Serafino was the most stable of all the cultivars analyzed; at the same time, it was the cultivar that was characterized by the largest amount of DF and its components in the grain. The AMMI analysis of rye bioactive compounds reveals critical insights into genotype × environment interactions, paving the way for targeted breeding and sustainable cultivation strategies. The obtained results indicate the possibility of using hybrid rye cultivars as a source of DF, which may be an important element in preventing the growing number of diet-related diseases in society. In addition, knowledge of the presented features and their variability under the influence of environmental changes are important tools that can be used by plant breeders, scientists, and food technologists.

## Figures and Tables

**Table 1:** Mean values and analysis of variance (ANOVA) F-statistics and Tukey’s homogenous groups of dietary fiber and its components (g kg^−1^ DW) and alkylresorcinols (mg kg^−1^) content for main and interaction effects.

Effects	I-NSP	S-NSP	NSP	Lignin	DF	AX	β-Glucan	ARR
Cultivars								
KWS Binntto	86.5 ^a^	42.4 ^c^	128.9 ^ab^	24.4 ^ab^	153.3 ^ab^	71.6 ^a^	21.5 ^a^	1037 ^a^
KWS Serafino	84.2 ^b^	45.7 ^a^	129.9 ^a^	24.5 ^a^	154.4 ^a^	72.4 ^a^	21.8 ^a^	993 ^b^
Dan Rubin	83.9 ^b^	43.7 ^b^	127.6 ^b^	24.0 ^bcd^	151.6 ^b^	71.6 ^a^	20.9 ^b^	1013 ^ab^
Rye NN	84.4 ^b^	43.3 ^b^	127.7 ^b^	23.9 ^cd^	151.7 ^b^	72.6 ^a^	20.2 ^c^	961 ^c^
F statistics	13.7 **	41.9 **	6.8 **	4.8 **	9.3 **	3.8 ^n.s.^	40.2 **	19.6 **
Years								
2016/2017	75.6 ^b^	45.9 ^a^	121.5 ^c^	26.9 ^a^	148.5 ^c^	69.8 ^c^	20.1 ^c^	956 ^b^
2017/2018	89.8 ^a^	44.7 ^b^	134.5 ^a^	23.6 ^b^	158.0 ^a^	75.0 ^a^	21.3 ^b^	945 ^b^
2018/2019	88.9 ^a^	40.7 ^c^	129.6 ^b^	22.2 ^c^	151.7 ^b^	71.4 ^b^	21.9 ^a^	1103 ^a^
F statistics	826.4 **	222.1 **	334.0 **	496.8 **	162.7 **	131.2 **	88.5 **	194.7 **
Locations								
Boguszyn	84.9 ^b^	42.5 ^b^	124.4 ^b^	24.2 ^ab^	151.7 ^b^	71.8 ^bc^	21.7 ^a^	969 ^d^
Krzyżewo	84.5 ^b^	42.6 ^b^	127.1 ^b^	24.3 ^ab^	151.4 ^b^	70.7 ^cd^	20.9 ^b^	989 ^cd^
Prusim	87.1 ^a^	44.9 ^a^	132.0 ^a^	23.9 ^c^	155.9 ^a^	74.9 ^a^	20.6 ^b^	1014 ^ab^
Walewice	82.7 ^c^	44.6 ^a^	127.3 ^b^	26.7 ^a^	152.0 ^b^	70.4 ^d^	21.9 ^a^	1005 ^ab^
Wyczechy	84.6 ^b^	44.1 ^a^	128.8 ^b^	24.0 ^c^	152.8 ^b^	72.4 ^b^	20.5 ^b^	1027 ^a^
F statistics	19.5 **	23.1 **	20.1 **	4.5 **	14.4 **	35.5 **	27.2 **	7.59 **
Interactions (F-statistics)								
Year × Location	54.1 **	26.5 **	25.7 **	41.4 **	29.0 **	22.7 **	22.0 **	8.52 **
Year × Cultivar	6.9 **	29.6 **	9.5 **	16.1 **	10.2 **	13.9 **	7.6 **	7.6 **
Location × Cultivar	19.3 **	8.8 **	14.8 **	40.5 **	17.6 **	8.0 **	9.5 **	5.7 **
Year × Cultivar × Location	15.2 **	11.0 **	11.3 **	20.9 **	9.0 **	7.3 **	12.9 **	4.5 **

Note: **—significant at *p* ≤ 0.01; mean values with the same letters do not differ significantly; n.s.—not significant; I-NSP, insoluble fraction of non-starch polysaccharides; S-NSP, soluble fraction of non-starch polysaccharides; NSP, non-starch polysaccharides; DF, dietary fiber; AX, arabinoxylans; ARR, alkylresorcinols.

**Table 2:** Participation of variation sources in the total variance observed for analyzed individual variables (%) and F-statistics determined based on the AMMI analysis.

Parameter	Genotype (G)	Fst.	Environment (E) ^A^	Fst.	G×E Interaction	Fst.	Random Error
I-NSP	1.4	16.2 **	74.5	105.0 **	22.0	18.0 **	1.3
S-NSP	8.5	55.1 **	50.5	31.1 **	37.0	17.2 **	2.3
NSP	1.3	10.2 **	62.0	34.0 **	32.8	18.1 **	1.9
Lignin	0.4	5.3 **	39.8	72.7 **	58.0	52.0 **	1.2
DF	2.3	12.3 **	51.7	25.3 **	40.9	15.4 **	2.9
AX	1.1	7.0 **	57.9	17.8 **	35.0	15.4 **	2.4
β-glucan	10.9	41.7 **	41.6	29.8 **	42.2	11.6 **	3.9
ARR	7.1	37.7 **	58.9	14.3 **	26.7	10.1 **	2.8

Note: **—significant at *p* ≤ 0.01; ^A^—Cultivars in locations; I-NSP, insoluble fraction of non-starch polysaccharides; S-NSP, soluble fraction of non-starch polysaccharides; NSP, non-starch polysaccharides; DF, dietary fiber; AX, arabinoxylans; ARR, alkylresorcinols; Fst., F-statistic.

## Data Availability

The data presented in this study are available on request from the corresponding author due to privacy.

## Abbreviations

The following abbreviations are used in this manuscript:

AACC	American Association of Cereal Chemists
AMMI	Additive Main effects and Multiplicative Interaction model
ANOVA	Analysis of variance
AOAC	Association of Official Analytical Chemists
ARR	Alkylresorcinols
AX	Arabinoxylans
CAC	Codex Alimentarius Commission
Dan. Rubin	Dańkowskie Rubin
DF	Dietary fiber
DW	Dry weight
Fst.	F statistic
IDF	Insoluble fraction of dietary fiber
I-NSP	Insoluble fraction of non-starch polysaccharides
IPC	Interaction principal component
NCPs	Non-cellulose polysaccharides
NSPs	Non-starch polysaccharides
SDF	Soluble fraction of dietary fiber
S-NSPs	Soluble fraction of non-starch polysaccharides

## References

[1] Brzozowski L.J., Szuleta E., Philips T.D., Van Sanford D.A., Clark A.J. (2023). Breeding cereal rye (*Secale cereale*) for quality traits. Crop Sci..

[2] Klimek-Kopyra A., Bacior M., Neugschwandtner R. (2023). Hybrid rye (*Secale cereale* L.) as a good crop component to enhance yield stability in a winter cereal mixture. Acta Agrobot..

[3] Miedaner T., Lauenstein S., Lieberherr B. (2025). Comparison traits under less favourable environmental conditions and two input levels. Agriculture.

[4] Wang Y., Mette M.F., Miedaner T., Gottwald M., Wilde P., Reif J.C., Zhao Y. (2014). The accuracy of prediction of genomic selection in elite hybrid rye populations surpasses the accuracy of marker-assisted selection and is equally augmented by multiple field evaluation location and test years. BMC Genom..

[5] Kucerova J. (2009). Effects of location and year technological quality and pentosan content in rye. Czech J. Food Sci..

[6] Linina A., Kunkulberga D., Kronberga A., Locmele I. (2019). Winter rye grain quality of hybrid and population cultivars. Agron. Res..

[7] Bach Knudsen K.E., Nørskov N.P., Bolvig A.K., Hedemann M.S., Lærke H.N. (2017). Dietary fibers and associated phytochemicals in cereals. Mol. Nutr. Food Res..

[8] Delcour J.A., Aman P., Courtin C.M., Hamaker B.R., Verbeke K. (2016). Prebiotics, fermentable dietary fiber, and health claims. Adv Nutr..

[9] Dynkowska W. (2020). Rye (*Secale cereale* L.) arabinoxylans: Molecular structure, physicochemical properties and their resulting pro-health effects. Plant Breed. Seed Sc..

[10] Jones J.M. (2014). Codex-aligned dietary fiber definitions help to bridge the ‘fiber gap’. Nutr. J..

[11] McCleary B.V., De Vries J., Rader J.I., Cohen G., Prosky L., Mugford D.C., Champ M., Okuma K. (2011). Collaborative study report: Determination of insoluble, soluble and total dietary fiber (Codex Definition) by an enzymatic-gravimetric method and liquid chromatography. AACC International Report. Cereal Food World.

[12] Jonsson K., Andersson R., Bach Knudsen K.E., Hallmans G., Hanhineva K., Katina K., Kolehmainen M., Kyrø C., Langton M., Nordlund E. (2018). Rye and health–Where do we stand and where do we go?. Trends Food Sci. Technol..

[13] Fraś A., Gołębiewska K., Gołębiewski D., Boros D. (2018). Dietary fibre in cereal grains–A review. Plant Breed. Seed Sci..

[14] Galisteo M., Durate J., Zarzuelo A. (2008). Effects of dietary fibres on disturbances clustered in the metabolic syndrome. J. Nut. Biochem..

[15] Dikeman C.L., Fahey G.C. (2006). Viscosity as related to dietary fibre: A Review. Crit. Rev. Food Sci. Nutr..

[16] Ross A.B., Shepherd M.J., Schüpphaus M., Sinclair V., Alfaro B., Kamal-Eldin A., Aman P. (2003). Alkylresorcinols in cereals and cereal products. J. Agric. Food Chem..

[17] Horikawa K., Hashimoto C., Kikuchi Y., Makita M., Fukudome S.I., Okita K., Wada N., Oishi K. (2017). Wheat alkylresorcinols reduce micellar solubility of cholesterol in vitro and increase cholesterol excretion in mice. Nat. Prod. Res..

[18] Oishi K., Yamamoto S., Itoh N., Nakao R., Yasumoto Y., Tanaka K., Kikuchi Y., Fukudome S., Okita K., Ishikawa Y. (2015). Wheat alkylresorcinols suppress high-fat, high-sucrose diet-induced obesity and glucose intolerance by increasing excretion in male mice. J. Nutr..

[19] Boros D., Fraś A., Gołębiewska K., Gołębiewski D., Paczkowska O., Wiśniewska M. (2015). Nutritional Value and Prohealthy Properties of Cereal and Rapeseed Varieties Approved for Cultivation in Poland.

[20] Bach Knudsen K.E. (2014). Fiber and nonstarch polysaccharide content and variation in common crops used in broiler diets. Poult. Sci..

[21] Arczewska-Włosek A., Swiątkiewicz S., Bederska-Łojewska D., Orczewska-Dudek S., Szczurek W., Boros D., Fras A., Tomaszewska E., Dobrowolski P., Muszyński S. (2019). The efficiency of xylanase in broiler chickens fed with increasing dietary level of rye. Animals.

[22] Kołodziejczyk P., Makowska A., Pospieszna B., Michniewicz J., Paschke H. (2018). Chemical and nutritional characteristics of high-fibre rye milling fractions. Acta Sci. Pol. Technol. Aliment..

[23] Bieniek A., Buksa K. (2023). Properties and functionality of cereal non-starch polysaccharides in breadmaking. Appl. Sci..

[24] Buksa K., Ziobro R., Nawotna A., Praznik W., Gambuś H. (2012). Isolation, modification and characterization of soluble arabinoxylan fractions from rye grain. Eur. Food Res. Technol..

[25] Cyran M., Łapiński B. (2006). Physico-chemical characteristics of dietary fibre fractions in the grains of tetraploid and hexaploidy triticales: A comparison with wheat and rye. Plant Breed. Seed Sci..

[26] Wood P.J. (2007). Cereal β-glucan in diet and health. J. Cereal. Sci..

[27] Comino P., Collins H., Lahnstein J., Beahan C., Gidley M.J. (2014). Characterization of soluble and insoluble cell wall fractions from rye, wheat and hull-less barley endosperm flours. Food Hydrocoll..

[28] Nystrom L., Lampi A.M., Andersson A.A.M., Kamal-Eldin A., Gebruers K., Courtin C.M., Delcour J.A., Li L., Ward J.L., Fraś A. (2008). Phytochemicals and dietary fiber components in rye varieties in the Healthgrain diversity screen. J. Agric. Food Chem..

[29] Ikram A., Saeed F., Afzaal M., Abdullah M., Niaz B., Asif Khan M., Hussain M., Adnan Nasir M., Siddeeg A. (2022). Camparative study of biochemical properties, anti-nutritional profile, and antioxidant activity of newly developed rye variants. Int. J. Food Prop..

[30] Bederska-Łojewska D., Świątkiewicz S., Arczewska-Włosek A., Schwarz T. (2017). Rye nonstarch polysaccharides: Their impact on poultry intestinal physiology, nutrients digestibility and performance indices—A review. Ann. Anim. Sci..

[31] Milczarek A., Osek M., Skrzypek A. (2020). Effectiveness of using a hybrid rye cultivar in feeding broiler chickens. Can. J. Anim. Sci..

[32] Sułek A., Cacak-Pietrzak G., Studnicki M., Grabiński J., Nieróbca A., Wyzińska M., Różewicz M. (2024). Influeance of nitrogen fertilization level and weather conditions on yield and quantitative profile of anti-nutritional compounds in grain of selected rye cultivars. Agriculture.

[33] Grabiński J., Sułek A., Wyzińska M., Stuper-Szablewska K., Cacak-Pietrzak G., Nieróbca A., Dziki D. (2021). Impact of genotype, weather conditions and production technology on the quantitative profile of anti-nutritive compounds in rye grains. Agronomy.

[34] Kulawinek M., Jaromin A., Kozubek A., Zarnowski R. (2008). Alkylresorcinols in selected Polish rye and wheat cereals and whole-grain products. J. Agric. Food Chem..

[35] AACC (2003). Approved Methods of the AACC.

[36] AOAC (1995). Official Methods of Analysis of AOAC.

[37] Englyst H.N., Cummings J.H. (1984). Simplified method for the measurement of total non-starch polysaccharides by gas-liquid chromatography of constituent sugars as alditol acetates. Analyst.

[38] Theander O., Westerlund E.A. (1986). Studies on dietary fiber. 3. Improved procedures for analysis of dietary fiber. J. Agric. Food Chem..

[39] Tłuścik F., Kozubek A., Mejbaum-Katzenellenbogen W. (1981). Alkylresorcinols in rye (*Secale cereale* L.) grains. VI: Colorimetric micromethod for the determination of alkylresorcinols with the use of diazonium salt, Fast Blue B. Acta Soc. Bot. Polon..

[40] Cloud Software Group, Inc (2023). Data Science Workbench, Ver. 14.

