# Correction: Fraś et al. Characteristics of the Content and Variability of Dietary Fiber Components and Alkylresorcinols of Rye Grain (*Secale cereale* L.). *Molecules* 2025, *30*, 2994

**DOI:** 10.3390/molecules30183834

**Published:** 2025-09-22

**Authors:** Anna Fraś, Magdalena Wiśniewska, Dariusz R. Mańkowski, Marlena Gzowska

**Affiliations:** Plant Breeding and Acclimatization Institute—National Research Institute, Radzików, 05-870 Błonie, Poland; a.fras@ihar.edu.pl (A.F.); m.wisniewska@ihar.edu.pl (M.W.); d.mankowski@ihar.edu.pl (D.R.M.)

## Acknowledgments

In the published paper [1], the authors inadvertently omitted the Acknowledgments section regarding participation in the research process. The authors gratefully thank Danuta Boros for developing the concept and coordinating Task 3 of the ENERGYFEED project entitled “Determination and comparative analysis of endogenous anti-nutrients in grains of different cereal species and varieties and fixed mixtures intended to be used in animal trials”, in which the results of the described chemical analyses were obtained.

The authors state that the scientific conclusions are unaffected. This correction has been approved by the Academic Editor. The original publication has also been updated.
